# MicroRNA-377-3p inhibits hepatocellular carcinoma growth and metastasis through negative regulation of CPT1C-mediated fatty acid oxidation

**DOI:** 10.1186/s40170-021-00276-3

**Published:** 2022-01-20

**Authors:** Ting Zhang, Yanan Zhang, Jie Liu, Yan Ma, Qinong Ye, Xinlong Yan, Lihua Ding

**Affiliations:** 1 Department of Medical Molecular Biology, Beijing Institute of Biotechnology, Beijing, China; 2 Faculty of Environment and Life, Beijing University of Technology, Beijing, China; 3 The Brain Science Center, Beijing Institute of Basic Medical Sciences, Beijing, China; 4 No. 970 Hospital of Joint Logistics Support Force of PLA, Yantai, China

**Keywords:** Hepatocellular carcinoma, miR-377-3p, CPT1C, Fatty acid oxidation, Tumor growth, Metastasis

## Abstract

**Background:**

Altered lipid metabolism is closely related to the occurrence and development of hepatocellular carcinoma (HCC). Carnitine palmitoyltransferase 1C (CPT1C) is a member of CPT1 family and plays a key role in cancer development and progression. However, how microRNAs (miRNAs) regulate CPT1C-mediated fatty acid transport and oxidation remains to be elucidated.

**Methods:**

Oil Red O staining, mitochondrial, and lipid droplets immunofluorescence staining were used to detect the functions of miR-377-3p and CPT1C in fatty acid oxidation. Colocalization of palmitate and mitochondria was performed to investigate the function of miR-377-3p and CPT1C in fatty acid transport into mitochondria. Fatty acid oxidation (FAO) assay was used to detect the function of miR-377-3p and CPT1C in FAO. Cell proliferation, migration and invasion assays and animal experiments were used to evaluate the role of miR-377-3p/CPT1C axis in HCC progression in vitro and in vivo. Immunofluorescence staining was used to identify the clinical significance of miR-377-3p and CPT1C in HCC patients.

**Results:**

MiR-377-3p inhibits CPT1C expression by targeting its 3’-untranslated region. Through repression of CPT1C, miR-377-3p suppresses fatty acid oxidation by preventing fatty acid from entering into mitochondria and decreasing ATP production in HCC cells. Inhibiting fatty acid oxidation abolishes the ability of miR-377-3p/CPT1C axis to regulate HCC proliferation, migration, invasion and metastasis in vitro and in vivo. In HCC patients, CPT1C is significantly upregulated, and miR-377-3p expression and lipid droplets are negatively correlated with CPT1C expression. High expression of miR-377-3p and CPT1C predict better and worse clinical outcomes, respectively.

**Conclusions:**

We uncover the key function and the relevant mechanisms of the miR-377-3p/CPT1C axis in HCC, which might provide a potential target for the treatment of HCC.

**Supplementary Information:**

The online version contains supplementary material available at 10.1186/s40170-021-00276-3.

## Background

Liver cancer is one of the most common malignant tumors in the world, and its morbidity and mortality are still rising year by year [1–3]. Seventy-five to 85% of primary liver cancer is defined as hepatocellular carcinoma (HCC). The occurrence and development of HCC is a complex process of multi-factor, multi-stage, and multi-channel regulation [4, 5]. Since HCC is almost asymptomatic in the early stage, most patients are diagnosed at the advanced stage, which presents great difficulties in the treatment of HCC. Although some HCC-related risk factors are relatively clear, such as viral hepatitis, liver cirrhosis, and alcohol abuse [6, 7], the molecular mechanism underlying the development and progression of HCC remains largely unknown. Thus, further understanding of HCC tumorigenesis needs to be clarified.

Recent studies demonstrate that altered lipid metabolism, which is among the most vital metabolic alterations in cancer, plays a critical role in tumor occurrence and development [8–11]. Continuous fatty acid oxidation (FAO) is frequently activated in cancer cells for producing extra energy, thus maintaining the rapid cell proliferation [11–14]. Several enzymes have been identified to promote FAO in solid tumors. Carnitine palmitoyltransferase 1 (CPT1), a key enzyme in FAO, is a rate-limiting FAO enzyme that catalyzes the acylation of long chain fatty acids and their entries into mitochondria for β-oxidation [15, 16]. CPT1 has three isoforms, including CPT1A, CPT1B, and CPT1C. As the last identified member of the CPT1 family, the location and carnitine acyltransferase activity of CPT1C remains controversial. Sierra et al. indicated that CPT1C predominantly located in endoplasmic reticulum and had carnitine palmitoyltransferase activity [17], while Wang et al. demonstrated that CPT1C localized in mitochondria in PANC-1 cells [18], and biochemical studies failed to show palmitoyltransferase activity for CPT1C [19]. CPT1C is highly expressed in various types of tumors [20], regulates cell proliferation and senescence in MDA-MB-231 and PANC-1 cells [21], and promotes gastric cancer progression [22]. CPT1C has been reported to take an active role in cancer development and progression [15, 23, 24]. However, the role of CPT1C in HCC is unknown.

MicroRNAs (miRNAs) are small single-stranded non-coding RNA molecules containing approximately 22 nucleotides, which function in RNA silencing and post-transcriptional regulation of gene expression through binding to 3′-untranslated region (UTR) of targeted mRNA [25, 26]. Emerging evidence demonstrates that dysregulation of miRNA leads to alteration of lipid metabolism and takes an active role in various human cancers. MiR-22 directly targets ACLY to suppress *de novo* lipid synthesis and inhibit tumor growth and metastasis in osteosarcoma, prostate cancer, cervical cancer and lung cancer [27]. MiR-148a targets HMGCR, PGC1α and SIRT7 to regulate hepatic lipid metabolism and hepatocarcinogenesis in mice [28]. In breast cancer, miR-27b affects mitochondrial oxidation by targeting PDHX, thereby promoting breast cancer progression [29]. However, whether there are miRNAs targeting CPT1C to regulate its expression and thus modulate lipid metabolism in HCC remains unclear.

In this study, we identified miR-377-3p as a key regulator of CPT1C expression and lipid metabolism. MiR-377-3p inhibited CPT1C expression by targeting its 3′-UTR and suppressed fatty acid β-oxidation, leading to repression of HCC proliferation, migration, invasion, and metastasis in vitro and in vivo. The CPT1C-mediated fatty acid β-oxidation played a critical role in miR-377-3p inhibition of HCC growth and metastasis, which supports the miR-377-3p/CPT1C axis as a novel target for the treatment of HCC.

## Methods

### Cell lines and cell culture

Human hepatocellular carcinoma cell lines HepG2 and MHCC97H, 3T3-L1 preadipocytes and human embryonic kidney cell line HEK-293T were purchased from American Type Culture Collection (ATCC). All cells were tested for mycoplasma contamination and cultured in Dulbecco’s modified Eagle’s medium (DMEM, Gibco) containing 10% fetal bovine serum (FBS, TIANHANG) and 1% Penicillin-Streptomycin Solution 100X (BioMed), at 37 °C with 5% CO_2_ in humidified air.

### Plasmids, transfection, and lentiviral infection

FLAG-labeled CPT1C eukaryotic expression vector was purchased from Sino Biological. The lentiviral vector expressing the fragment of CPT1C shRNA was cloned into the pSIH-H1-Puro (System Biosciences). The CPT1C shRNAs sequence was shown in Table S1. For lentiviral infection, HEK-293T cells were co-transfected with recombinant lentiviral vector and pPACK Packing Plasmid Mix (System Biosciences) using Megatran reagent (Origene) to obtain lentiviruses. Lentiviruses were used to infect HCC cells in the presence of polybrene (10 μg/ml). The successfully infected cells were screened with puromycin (1 μg/ml) and stable cell lines were obtained. MiR-377-3p mimics and inhibitors were purchased from JTS scientific. The sequences were shown in Table S1. Lipofectamine 3000 and Lipofectamine RNAiMAX reagents for transfection were performed according to the manufacturer’s instruction (Invitrogen). BODIPY 493/503 was obtained from Invitrogen. Specific anti-CPT1A (66039-1-Ig), specific anti-CPT1B (22170-1-AP), specific anti-CPT1C (66072-1-Ig), and specific anti-FASN (10624-2-AP) were purchased from Proteintech; specific anti-CPT1C (PA5-98783) was purchased from Thermo Scientific; specific anti-ACLY (AF1747) and specific anti-CD36 (AF6414) were purchased from beyotime; and β-actin antibody (sc-47778) and anti-mouse horseradish peroxidase (HRP)-conjugated IgG (sc-2748) were purchased from Santa Cruz Biotechnology.

### In situ hybridization of miRNA and immunohistochemistry

Ninety paired HCC tumor and adjacent normal tissues were obtained from Chinese PLA General Hospital, with the informed consent of patients and the hospital’s approval of the experiment. The follow-up data of 72 patients were available. To detect the expression level of miR-377-3p, miRNA in situ hybridization (MISH) on paraffin tissue sections with probes specific for human miR-377-3p was performed according to the manufacturer’s instructions (Exonbio). Digoxin was labeled to both 5′ and 3′ ends of the probe. The miRNA signal was amplified by TSA Plus Cyanine 5 system (PerkinElmer). The sequences of the miR-377-3p probe and U6 positive control were shown in Table S1. Immunohistochemistry (IHC) of formalin-fixed paraffin-embedded samples was performed as described previously [30]. Mouse anti-CPT1C was used at dilutions of 1:200 for IHC. BODIPY 493/503 (1 μg/ml) was used to stain lipid droplets in the samples. The fluorescence intensity and percentage of positive cells were counted and calculated by ImageJ software. The expression of miR-377-3p and CPT1C and lipid droplet level were assessed by H score, which was determined by multiplying the percentage of stained cells (0–100%) by the staining intensity (low, 1+; medium, 2+; strong, 3+). The median number was used as the cutoff value. We defined the score ≤ 1.3 and > 1.3 as low and high miR-377-3p respectively, and the score ≤ 1.4 and > 1.4 as low and high CPT1C, respectively.

### Luciferase reporter gene analysis

The 3′-UTR of human CPT1C gene was obtained by PCR. To introduce mutations into the predicted seed sequence of miR-377-3p target site, recombinant PCR was performed to generate Mut CPT1C 3′-UTR construct. The fragment was cloned into psiCHECK-2 dual luciferase miRNA target expression vector (Miaolingbio) to obtain the recombinant vectors CPT1C-wild-type (WT) and CPT1C-mutant (MUT). Primer sequences were shown in Table S1. To analyze the activity of reporter genes, cells seeded in 24-well plates were co-transfected with the psiCHECK-2 vector containing WT or MUT 3′-UTR of CPT1C driving the expression of luciferase and with either the miR-377 mimics or NC mimics. After transfection for 48 h, luciferase reporter gene activity was detected according to the manufacturer’s instructions (Vigorousbio).

### Western blotting

Transfected cells were lysed in RIPA lysis reagent containing protease inhibitor on ice for 30 min, and the protein concentration was determined using a BCA protein assay kit (Thermo Fisher Scientific). Equal amounts of protein were separated by SDS-PAGE, transferred onto the nitrocellulose membrane, incubated in 5% skimmed milk to block non-specific reaction, and then incubated overnight with primary antibodies at 4 °C. At last, the membranes were incubated with anti-mouse horseradish peroxidase (HRP)-conjugated IgG at room temperature for 1 h, and then detected by enhanced chemiluminescence detection (Thermo Fisher Scientific).

### RNA extraction and quantitative real-time PCR (qRT-PCR)

Total RNA was extracted from the collected cells using TRIzol reagent according to the manufacturer’s instructions (Invitrogen). For qRT-PCR, RNA was reverse transcribed to cDNA by using a Reverse Transcription Kit (Sbsbio). To detect the expression of miR-377-3p, reverse transcription was performed following the miRcute miRNA First-Strand cDNA synthesis Kit’ protocol (Tiangen). Real-time qPCR reactions were performed using Bio-Rad CFX96 with the miScript SYBR Green PCR Kit (Qiagen). The related primers were shown in Table S1. Relative changes of gene expression were calculated using the comparative Ct methods.

### Flow cytometry

After transfection for 48 h, cells were harvested by trypsinization, washed with PBS, and stained with BODIPY (1 μg/ml) according to the manufacturer’s instructions. After washing with PBS, the cells were resuspended into 300 μl PBS, and 1 × 10^4^ cells were collected and analyzed by Flow cytometry (BD Biosciences). Flow Jo V10 software was used to analyze the results.

### Oil Red O staining

Transfected cells grown on coverslips were seeded in 24-well plates. Cells were washed with PBS and fixed with 4% paraformaldehyde for 30 min, then cells were washed twice with PBS, and stained for 10 min in Oil Red O (Solarbio) working solution (stock solution/ddH2O, 3:2). At last, the cells were counter-stained with Harris hematoxylin for 20 s and sealed with glycerin. Images were obtained using a light microscope. The relative account of lipid droplet was analyzed with ImageJ software.

### Mitochondrial, CPT1C, and lipid droplets immunofluorescence staining

Immunofluorescence staining of mitochondrial and lipid droplets was performed according to manufacturer’s instructions. Briefly, after removing DMEM in the 24-well plate with cells grown on coverslips, Mito-Tracker Red CMXROS (Beyotime, C1035) working solution was added, and then incubated in the dark at 37 °C for 25 min. After washing with PBS, cells were fixed with 4% paraformaldehyde for 30 min. Cells were washed twice with PBS and then stained in the dark for 15 min at 37 °C with BODIPY 493/503 (1 μg/ml). For CPT1C immunofluorescence assay, the cells were incubated with anti-CPT1C antibody at 4 °C overnight, and then Alexa Fluor 488 conjugate secondary antibody (Abcam, ab150113) was applied at room temperature for 1 h. At last, nuclei were stained using DAPI (5 μg/ml) for 5 min before sealing. Images were obtained using a fluorescence microscope.

### Uptake of CY3-labeled palmitate

Tracing of CY3-labeled palmitate and staining of mitochondria were performed according to manufacturer’s instructions. Cells were plated in 24 wells with coverslips and incubated with CY3-labeled palmitate (2.5 μM) for 24 h. After the treatment, the culture medium was removed and the cells incubated with Mito-Tracker Green (Beyotime, C1048) working solution in the dark at 37 °C for 25 min. The nucleus was stained with Hoechst 33342 (10 μg/mL) (Beyotime, C1028) for 10 min. Images were obtained using a fluorescence microscope.

### Fatty acid oxidation (FAO) assay

Fatty acid oxidation assay was performed as described in the manual (Abcam, ab222944). Briefly, approximately 6 × 10^4^ cells per well were plated into 96-well plates. Cells treated with 2.5 μM carbonyl cyanide 4-(trifluoromethoxy) phenylhydrazone (FCCP) were used as positive control, and cells treated with 40 μM Etomoxir were used as negative control. Rates of FAO were calculated using slopes (m) from the linear portion of each profile as follows: FAO = m_untreated_ − m_Etomoxir_.

### ATP production assay

The ATP production was measured using a firefly luciferase-based ATP assay kit (Beyotime, S0026) according to the manufacturer’s instructions. Briefly, cells or tissues were fully lysed and centrifuged at 4 °C, 12,000×*g* for 5 min. The supernatants (20 μl) were mixed with 100 μL of ATP detection working dilution in Eppendorf tubes. Luminescence was measured by a luminometer.

### Cell proliferation

Cell proliferation was assessed using the CCK-8 Kit (Dojindo) and colony forming assay. For CCK8 assay, cells were plated in a 96-well plate at 1 × 10^3^/well. The proliferation was detected by CCK-8 for 5 times in interval of 24 h. Three replicates were detected for each time point. For colony forming assay, cells were seeded at 2 × 10^3^/well in 6-well plates. After 14 days, cells were fixed with 4% paraformaldehyde for 10 min and stained with 0.5% crystal violet solution for 20 min. Images were scanned, and colony numbers were quantified with ImageJ software.

### Invasion assay

For cell invasion assay, transfected cells in serum-free media were placed into the upper chamber of the 24-well transwell insert (8 μm pore size, Corning Costar), and the lower chamber was added with 700 μl of media with 20% FBS for invasion assay with Matrigel. After incubation of 16 h, the cells that had invaded through the membrane were washed with PBS, 4% paraformaldehyde fixation and 0.5% crystal violet staining were performed for 30 min, respectively.

### Migration assay

Wound healing assay and transwell assay without matrigel were applied for the detection of cell migration. For wound healing assay, cells were seeded into 6-well plates at the density of 90%. A 200 μl pipette tip was employed for straight scratch in each well center, followed by washing separated cells with PBS. Then, cells were continually cultured for 16 h, the speed of wound closure was measured and further analyzed for cell migration. The migration ability of cells was also detected with transwell assay without Matrigel as the above-mentioned invasion assay.

### Analysis of tumor growth and metastasis in vivo

All animal studies were approved by the Institutional Animal Care Committee of Beijing Institute of Biotechnology. A total of 1 × 10^7^ HepG2 cells infected with a lentivirus expressing either CPT1C shRNA or negative control were injected subcutaneously into the flank of BALB/c nude mice (4–6 weeks old, female, four groups, *n* = 6 per group). After 10 days of transplantation, miR-377-3p antagomir or NC antagomir (GenePharma) was directly injected into the implanted tumors at multipoint, 15 μg (in 50 μL PBS) per mouse, twice a week for eight times. Tumor size was monitored every 7 days for 42 days. The tumor volume was calculated using the following formula: volume = (length × width^2^)/2. After euthanasia, the tumors were excised and frozen in liquid nitrogen for further study.

For lung metastasis study, 1 × 10^6^ MHCC97H cells were injected into the lateral tail vein of BALB/c nude mice (4–6 weeks old, female, four groups, *n* = 4 per group). After 10 days of injection, miR-377-3p antagomir or NC antagomir was intravenously injected with 45 μg (in 100 μL PBS) per mouse for three consecutive days. Then, all the lungs were excised for metastatic foci analysis after euthanasia.

### Statistical analysis

All statistical analyses were performed using GraphPad Prism 7. The correlation between miR-377-3p expression and CPT1C expression, and between miR-377-3p expression and lipid droplet level, and between CPT1C expression and lipid droplet level were analyzed by Spearman correlation analysis, respectively. Results in this study were subjected to one-way analysis of variance or Student’s *t* test and were presented as mean ± SD. All experiments were conducted in at least triplicate. *p* < 0.05 was defined to indicate statistical significance.

## Results

### MiR-377-3p targets the CPT1C 3’-UTR

**Fig. 1:**
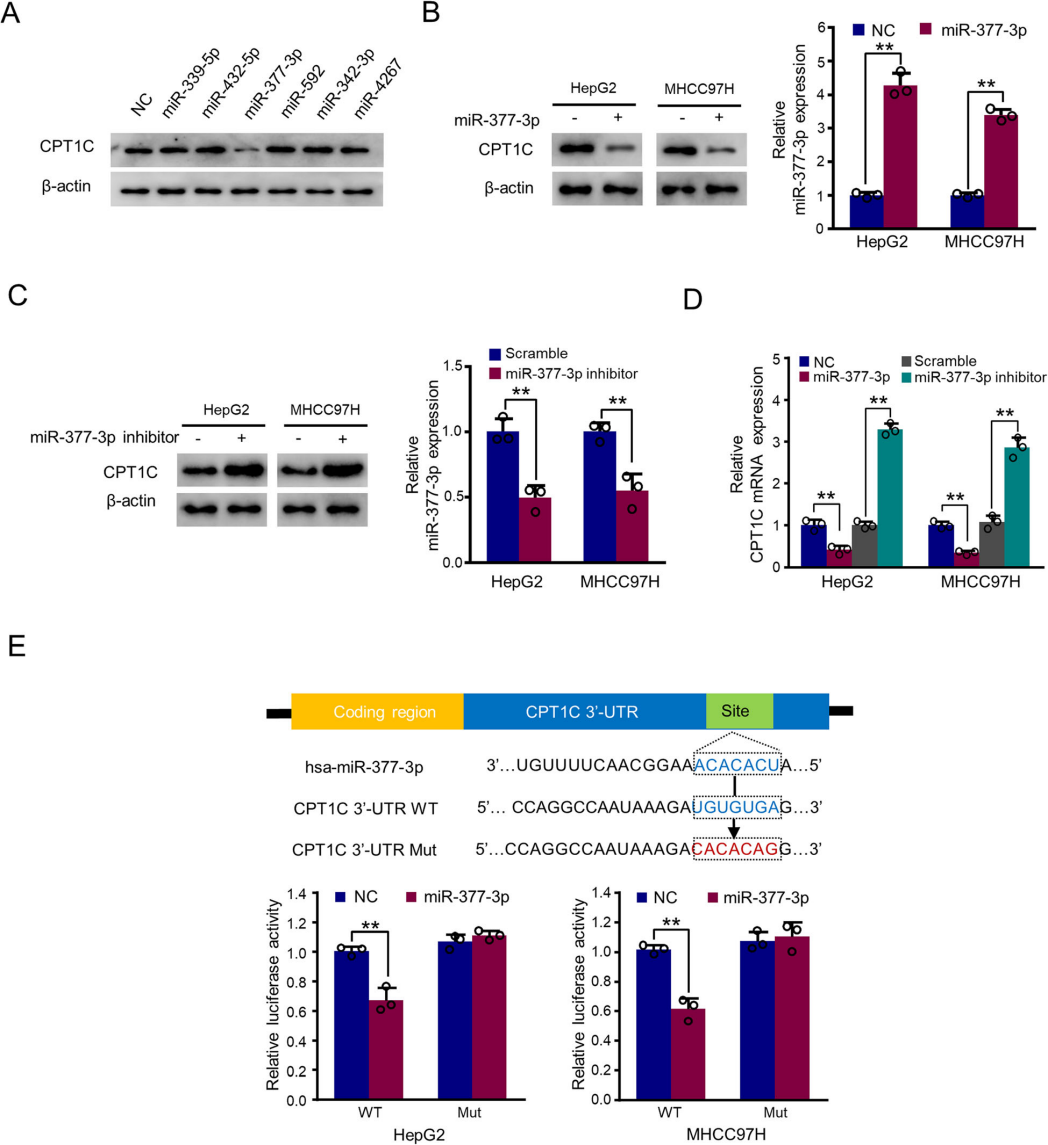
MiR-377-3p targets the 3’-UTR of CPT1C and inhibits its expression in HCC. **A** HEK-293T cells were transfected with negative control (NC) or mimics of candidate miRNAs as indicated. The representative western blot shows CPT1C expression. β-actin was used as a loading control for western blot. **B**, **C** Western blot analysis of CPT1C expression after transfected with (**B**) NC or miR-377-3p mimics, or (**C**) scramble or miR-377-3p inhibitor in HepG2 and MHCC97H cells. Histograms show relative CPT1C expression by RT-qPCR. The values of the control groups in HepG2 and MHCC97H cells were set to 1. **D** Levels of CPT1C mRNA in HepG2 and MHCC97H cells transfected as in (**B**) and (**C**). The values of the control groups in HepG2 and MHCC97H cells were set to 1. **E** Luciferase reporter gene assay was conducted in HepG2 and MHCC97H cells to compare CPT1C 3′-UTR activity among plasmids within wild-type (WT) or mutant (Mut) CPT1C 3′-UTR luciferase reporter with overexpression of miR-377-3p. The top panel indicates WT and Mut forms of putative miR-377-3p target sequences of CPT1C 3′-UTR. Blue font indicates the putative miR-377-3p binding sites within human CPT1C 3′-UTR. Red font indicates the mutations introduced into the CPT1C 3′-UTR. All values shown are mean ± SD (*n* = 3). **p < 0.01

Since CPT1C is reported as a brain-specific isoform, we first examined the expression of CPT1C in HCC cells and HEK-293T cells using mouse brain tissue and CPT1C expression vector as positive controls (Supplementary Figure 1A). Furthermore, immunofluorescence assay indicated that CPT1C colocalized with mitochondria in HCC cells (Supplementary Figure 1B). The specificity of CPT1C antibody was verified with CPT1C shRNA-expressing cells (Supplementary Figure 1C). Next, we screened potential miRNAs targeting CPT1C. Six miRNAs that might target CPT1C were predicted by TargetScan and miRanda databases, including miR-339-5p, miR-432-5p, miR-377-3p, miR-592, miR-342-3p, and miR-4267. Western blot analysis in HEK-293T cell showed that miR-377-3p significantly inhibited the expression of CPT1C (Fig. 1A). Therefore, miR-377-3p was selected for further study. Concordant with the results in HEK-293T cell, miR-377-3p mimics inhibited the expression of CPT1C in HepG2 and MHCC97H cells, while miR-377-3p had no effect on the expression of CPT1A and CPT1B (Fig. 1B and Supplementary Figure 1D). In contrast, the miR-377-3p inhibitor increased CPT1C expression in both two cell lines (Fig. 1C). In addition, miR-377-3p mimics down-regulated CPT1C mRNA level, while miR-377-3p inhibitor upregulated CPT1C mRNA expression (Fig. 1D). Furthermore, miR-377-3p mimics had no effect on the expression of de novo lipid synthesis-related enzymes (FASN and ACLY) and lipid up-taking fatty acid translocase (CD36) (Supplementary Figure 1E). In order to clarify how miR-377-3p regulates CPT1C expression in cancer cells, we transfected wild-type or mutant CPT1C 3′-UTR luciferase reporter and miR-377-3p into HepG2 and MHCC97H cells. The luciferase reporter assay showed that increased miR-377-3p expression significantly reduced CPT1C 3′-UTR activity, but produced a smaller or no change in the luciferase activity of mutant CPT1C 3′-UTR luciferase reporter (Fig. 1E). In conclusion, these results indicate that miR-377-3p negatively regulates CPT1C expression by binding to the 3′-UTR region of its mRNA.

### MiR-377-3p regulates FAO of lipid metabolism through CPT1C

**Fig. 2:**
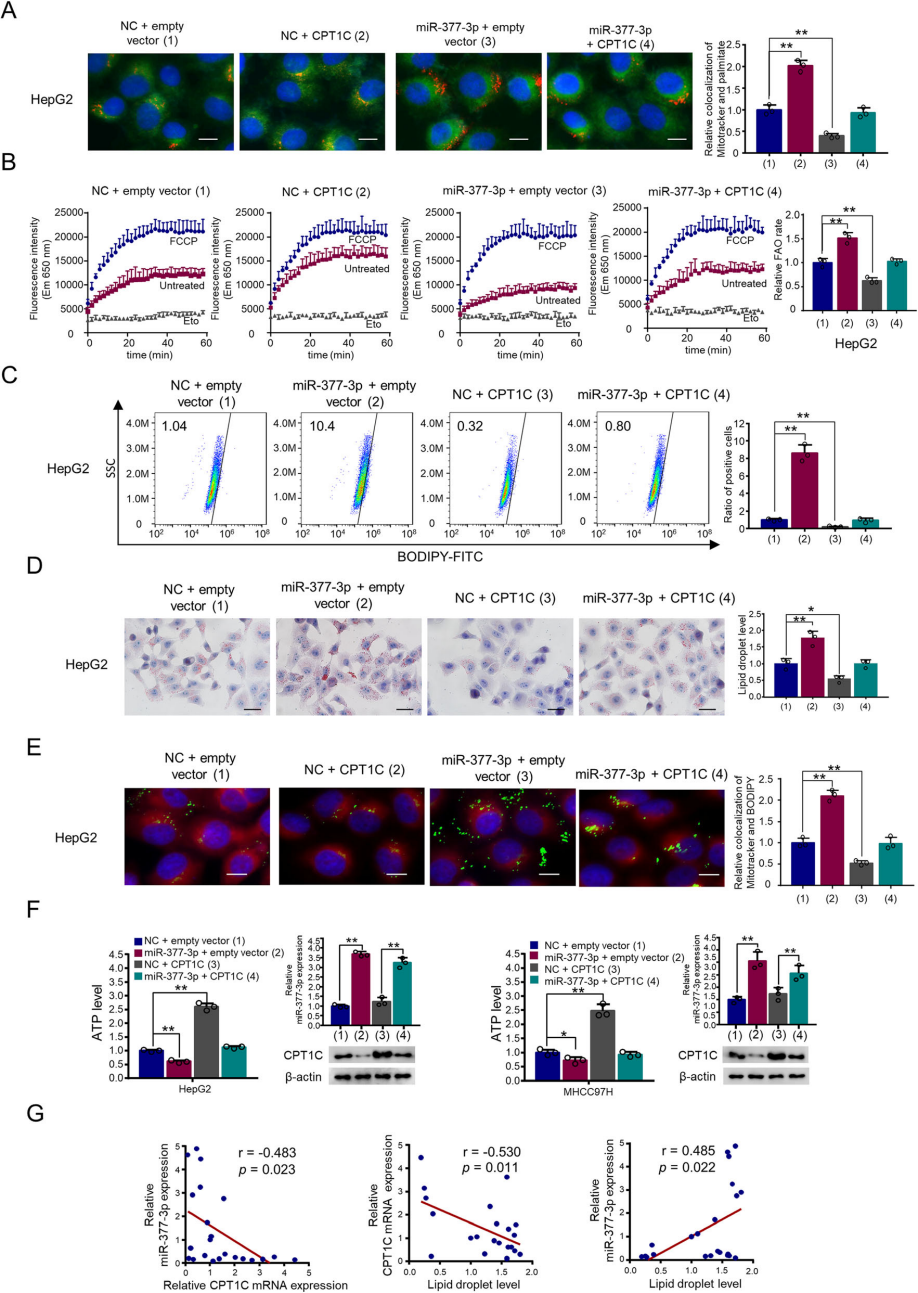
MiR-377-3p regulates FAO of lipid metabolism through CPT1C. **A** Representative images of CY3-labeled palmitate (red) and staining of mitochondria (green) in HepG2 cells transfected with CPT1C expression plasmid or miR-377-3p mimics or miR-377-3p mimics plus CPT1C expression plasmid. The colocalization was shown as yellow. Graphs at the right panel show relative colocalization number of mitochondria and CPT1C. Results shown are mean ± SD of 3 independent experiments. Scale bar 10 μm. **B** FAO assay of HepG2 cells transfected as in **A**. Cells treated with FCCP were used as positive control, and cells treated with Eto were used as negative control. Eto, Etomoxir. Graphs at the right panel show relative FAO rate. Data shown are mean ± SD of 3 independent experiments. **C** HepG2 cells were transfected as in **A** and stained with BODIPY 493/503 and then the lipid droplet content was determined by flow cytometry. Graphs at the right panel show relative ratio of positive cells. Data shown are mean ± SD of three independent experiments. **D** Cells were transfected as in **A** and stained with Oil Red O. Graphs at the right panel show relative LD content. Data shown are mean ± SD of three independent experiments. Scale bar 20 μm. **E** Representative images of colocalization between green BODIPY 493/503 (lipid droplets) and red Mito Tracker (mitochondria). The colocalization was shown as yellow. Graphs at the right panel show relative colocalization of mitochondria and LD. Data shown are mean ± SD of three independent experiments. Scale bar 10 μm. **F** ATP production was determined in HepG2 and MHCC97H cells transfected as in **A**. Representative western blot confirms the expression of CPT1C. RT-qPCR analysis indicates miR-377-3p expression. Data are mean ± SD (*n* = 3). *p < 0.05 and **p < 0.01. **G** Correlation between miR-377-3p expression, CPT1C expression and lipid droplet level in 22 cell lines. The *p* value was generated using Spearman’s rank correlation test

Since the function of CPT1C in transport of fatty acids into mitochondria for β-oxidation is still controversial, and CPT1C is the target gene of miR-377-3p, we investigated the function of miR-377-3p/CPT1C in fatty acid transport and β-oxidation. With tracing of CY3-labeled palmitate, we found that CPT1C enhanced the colocalization of palmitate with mitochondria in HCC cells, and miR-377-3p mimics inhibited the colocalization of palmitate with mitochondria (Fig. 2A and Supplementary Figure 2A). Importantly, miR-377-3p mimics almost abolished the CPT1C-mediated transport of palmitate into mitochondria, indicating that miR-377-3p inhibits CPT1C-mediated colocalization of palmitate with mitochondria (Fig. 2A and Supplementary Figure 2A). FAO analysis indicated that CPT1C enhanced FAO in HCC cells, whereas miR-377-3p mimics inhibited FAO. Importantly, miR-377-3p mimics almost abolished CPT1C-mediated FAO (Fig. 2B and supplementary Fig. 2B). To investigate the lipid droplet content with cytometry analysis, we used 3T3-L1 non-differentiated preadipocyte as negative control, and the differentiated 3T3-L1 adipocyte as positive control (Supplementary Figure 2C). Flow cytometry assay showed that compared to the control group, transfection of CPT1C decreased the lipid droplet content, while miR-377-3p mimics increased the lipid droplet content (Fig. 2C and supplementary Fig. 2D). Moreover, reexpression of CPT1C in the miR-377-3p-transfected cells reversed these effects in HepG2 and MHCC97H cells. Meanwhile, oil red O staining showed the same results (Fig. 2D and Supplementary Figure 2E). To assess the effect of miR-377-3p on FAO in HCC, we further analyze the differences in lipid transport to mitochondria between different groups. Mitochondria and lipid droplets were stained with Mito-Tracker Red CMXROS and BODOPY 493/503, respectively. The results showed that CPT1C overexpression increased transport of lipids into the mitochondria, miR-377-3p decreased transport of lipids into the mitochondria, and reexpression of CPT1C in the miR-377-3p-transfected cells reversed these effects (Fig. 2E and Supplementary Figure 2F). Further examination of ATP levels indicated the same trends (Fig. 2F). Conversely, miR-377-3p inhibitor reduced the amount of lipid droplets, increased the proportion of lipid droplets entering the mitochondria and increased the ATP level in HCC cells (Supplementary Figure 2G–J).

In order to further prove the correlation between miR-377-3p and CPT1C expression as well as the lipid droplet level, we detected 22 different cell lines together. The statistical results indicated that there was an inverse correlation between miR-377-3p and CPT1C, a similar result was observed between CPT1C expression and lipid droplet level (Fig. 2G). As expected, miR-377-3p expression and lipid droplet level were positively correlated (Fig. 2G). The expression of miR-377-3p, CPT1C and lipid droplets were shown in detail in Supplementary Figure 2K. In conclusion, these data suggest that miR-377-3p regulates FAO of lipid metabolism through CPT1C in HCC cells.

### MiR-377-3p suppresses proliferation, migration, and invasion through inhibition of CPT1C expression in HCC cells

**Fig. 3:**
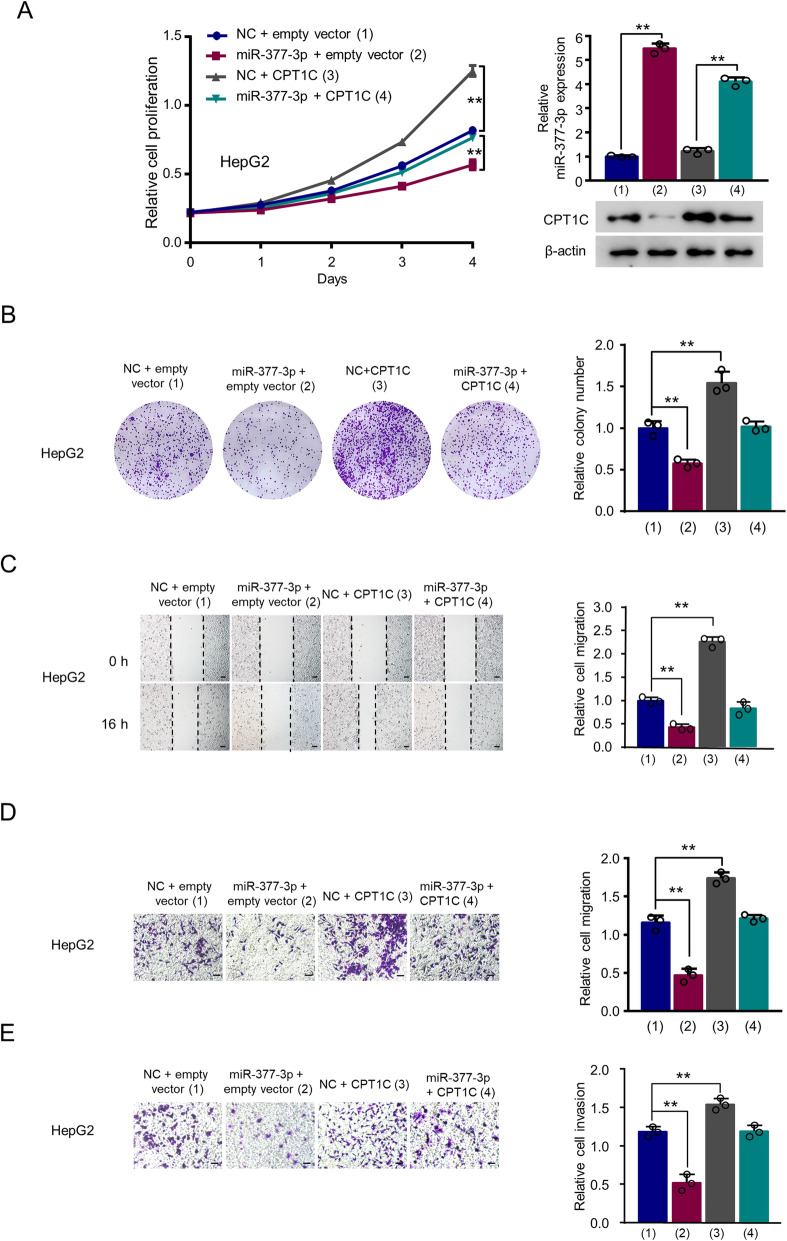
MiR-377-3p suppresses proliferation, migration and invasion through inhibition of CPT1C expression in HCC cells. **A** The proliferation curve of HepG2 cells transfected with CPT1C expression plasmid or miR-377-3p mimics or miR-377-3p mimics plus CPT1C expression plasmid. Western blot analysis shows CPT1C expression. RT-qPCR analysis indicates miR-377-3p expression. **B** Colony formation assay of HepG2 cells transfected as in A. Graphs at the right panel show relative colony number. Data shown are mean ± SD of 3 independent experiments. **C** Wound scratch healing assays of HepG2 cells after transfection as in **A**. Scale bar 50 μm. Histograms show relative cell migration. **D**, **E** Transwell assay of HepG2 cells transfected as in **A** without **D** or with **E** Matrigel. Graphs at the right panel show relative cell migration and invasion. The values of the control groups in HepG2 cells were set to 1. Data are mean ± SD (*n* = 3). **p < 0.01

Since miR-377-3p has been reported to be a tumor suppressor [31, 32], and CPT1C was a target gene of miR-377-3p, we further explored the effects of the miR-377-3p/CPT1C axis on HCC cells. CCK8 and colony formation assays showed that transfection of miR-377-3p decreased HCC cell proliferation, while CPT1C exhibited the opposite effects. Moreover, reexpression of CPT1C in miR-377-3p-transfected cells rescued the miR-377-3p-induced reduction of cell proliferation (Fig. 3A, B and Supplementary Figure 3A). Wound healing assay and transwell assay with or without matrigel demonstrated that miR-377-3p decreased the capability of HCC cell migration and invasion, while CPT1C overexpression increased the capability of HCC cells, and CPT1C overexpression abrogated the effect of miR-377-3p on HCC cell migration and invasion (Fig. 3C–E and Supplementary Figure 3B, C).

In contrast, miR-377-3p inhibitor promoted the proliferation, migration and invasion capability of HCC cells. CPT1C knockdown abolished the ability of miR-377-3p inhibitor to increase the proliferation, migration and invasion of HCC cells (Supplementary Fig. 3D–F). These results indicate that miR-377-3p suppresses proliferation, migration and invasion by repressing CPT1C expression in HCC cells.

### The miR-377-3p/CPT1C axis regulates proliferation, migration and invasion of HCC cells mainly through FAO

**Fig. 4:**
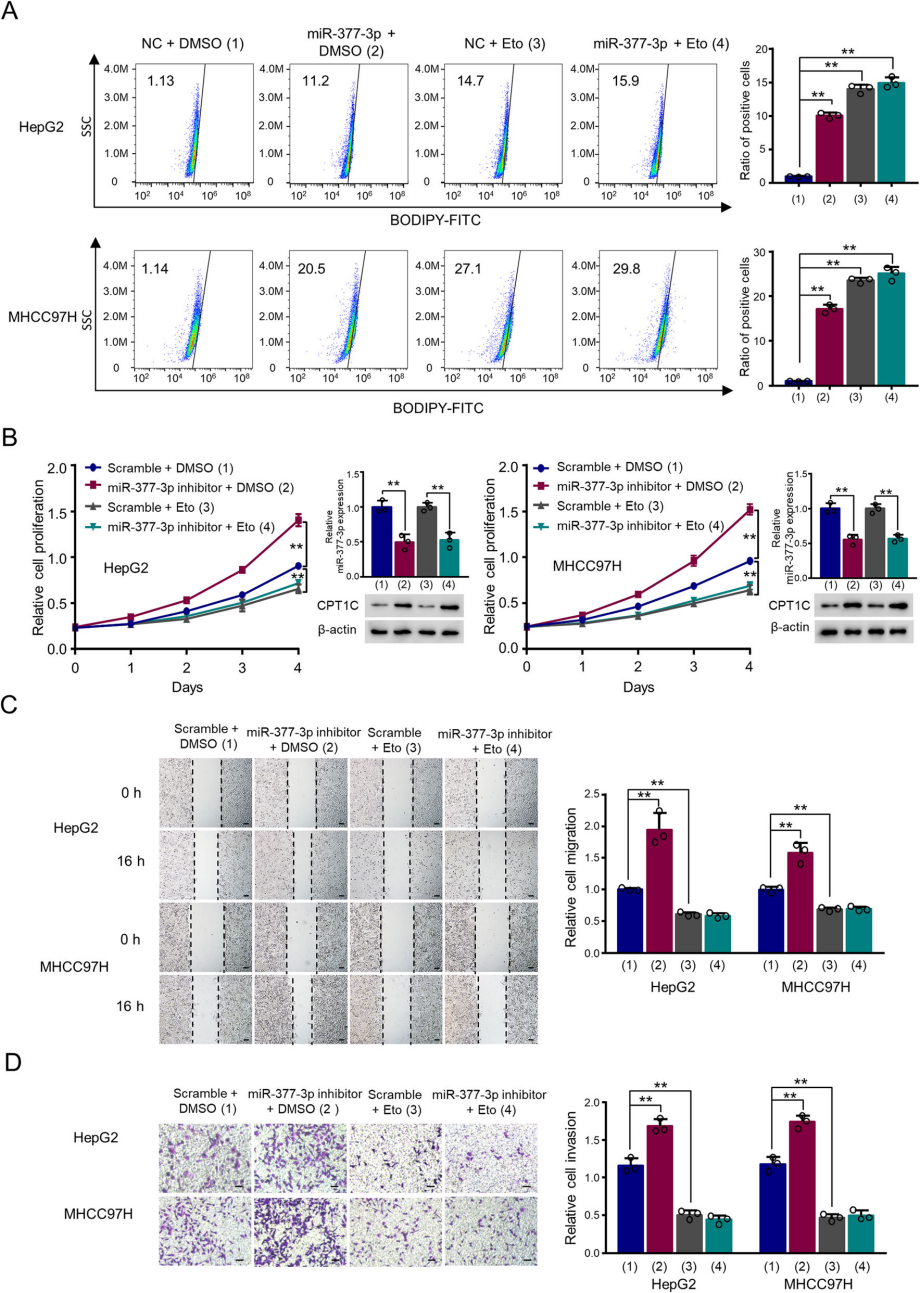
The miR-377-3p/CPT1C axis regulates the proliferation, migration, and invasion of HCC cells mainly through FAO. **A** HepG2 cells were transfected with CPT1C expression plasmid or empty vector and treated with indicated dose of Etomoxir. Cells were stained with BODIPY 493/503 and the lipid droplet content was determined by flow cytometry. Eto, Etomoxir. Graphs at the right panel show relative ratio of positive cells. Data shown are mean ± SD of 3 independent experiments. **B** The proliferation curve of HepG2 and MHCC97H cells transfected with miR-377-3p inhibitor or scramble and treated with Etomoxir (5 μM) as indicated. Western blot analysis shows CPT1C expression. RT-qPCR analysis indicates miR-377-3p expression. **C**, **D** Wound scratch healing (**C**) and invasion (**D**) assays of HepG2 and MHCC97H cells after transfection as in (**B**). Scale bar 50 μm. Histograms show relative cell migration and invasion. The values of the control groups in HepG2 and MHCC97H cells were set to 1. Data are mean ± SD (*n* = 3). **p < 0.01

Since miR-377-3p regulated FAO and cell proliferation, migration and invasion through CPT1C, we investigated whether miR-377-3p/CPT1C axis regulates these phenotypes through FAO. Firstly, the CPT1C-mediated lipid droplet content decrease was abolished by Etomoxir in a dose-dependent manner, a CPT1 inhibitor (Supplementary Fig. 4A). Knock-down of CPT1A or CPT1C enhanced the lipid droplet content, and Etomoxir treatment further increased the LD content, suggesting that Etomoxir promoted the LD content through inhibiting the CPT1A and CPT1C activity (Supplementary Fig. 4B). Importantly, Etomoxir abrogated miR-377-3p-mediated lipid droplet content increase (Fig. 4A). Next, miR-377-3p inhibitor-mediated enhancement of proliferation, migration and invasion were reversed by Etomoxir in HepG2 and MHCC97H cells (Fig. 4B–D). On the other hand, acetate can be converted to acetyl-CoA, which is the end product of FAO, we hypothesized that FAO could be rescued by acetate. Our results showed that miR-377-3p-induced suppression of cell proliferation, migration and invasion could be rescued by restoring FAO with acetate supplementation (Supplementary Figure 5A–C). These data indicate that miR-377-3p/CPT1C axis modulates HCC cell proliferation, migration and invasion mainly through FAO.

### The miR-377-3p/CPT1C axis regulates HCC tumor growth and metastasis in nude mice

**Fig. 5:**
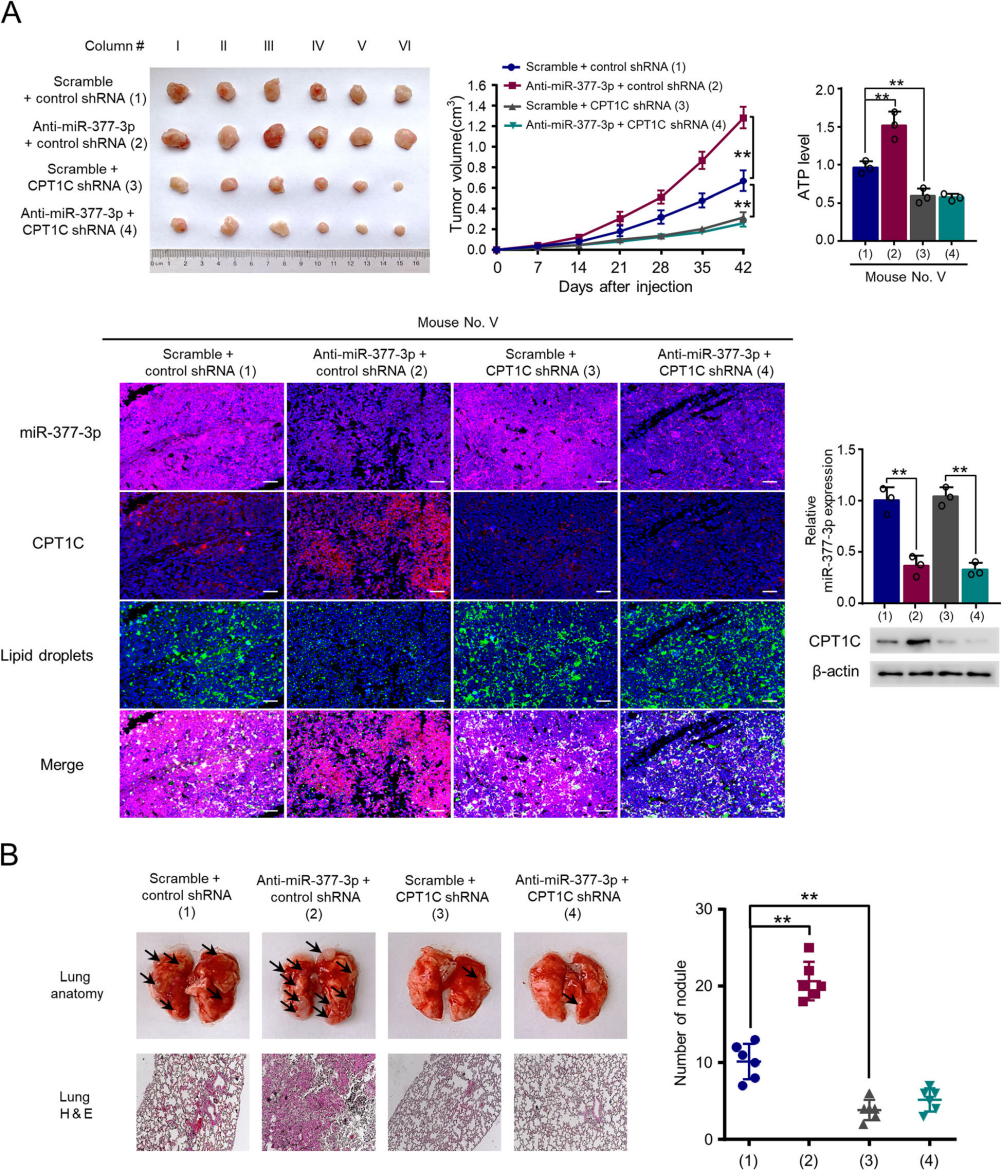
The miR-377-3p/CPT1C axis regulates HCC tumor growth and metastasis in nude mice. **A** HepG2 cells stably infected with lentivirus carrying CPT1C shRNA or control shRNA were treated with antagomir miR-377-3p (anti-miR-377-3p) or antagomir NC (scramble) and injected into nude mice as indicated. After 42 days, mice were euthanized to harvest tumors. Images of all xenograft tumors excised at day 42 were shown. The tumor growth curves were plotted. ATP level of tumor tissues (mouse no. V) was determined. MiR-377-3p expression in representative tumor tissues (mouse no. V) was examined by MISH and RT-qPCR, and CPT1C expression was determined by IHC and western blot, and lipid content was conducted by BODIPY 493/503, respectively. Tumor volumes are presented as means ± SD (*n* = 6). **p < 0.01 at day 42. Data shown are mean ± SD of quintuplicate measurements for ATP level and were repeated 3 times with similar results. **p < 0.01. **B** Representative lung tissues and H&E-stained sections of the lung tissues. The number of tumor nodules were shown (right panel). **p < 0.01

To define the role of the miR-377-3p/CPT1C axis in vivo, we established xenograft mouse model to evaluate the impact of the axis on HCC tumor growth. As expected, tumors derived from the CPT1C knockdown group were much smaller and those from the miR-377-3p inhibitor group were much bigger compared with the tumors in control group (Fig. 5A). Moreover, the ability of miR-377-3p inhibitor to promote the growth of cancer xenografts were abolished by CPT1C knockdown. ATP production and immunofluorescence staining assays of the tumor masses further confirmed that miR-377-3p repressed FAO through CPT1C (Fig. 5A). These data indicate that miR-377-3p suppresses tumor growth through CPT1C-mediated FAO.

Furthermore, we explored whether the miR-377-3p/CPT1C axis regulates HCC metastasis in nude mice. The results declared that miR-377-3p inhibitor enhanced lung metastasis of MHCC97H cells, while CPT1C knockdown inhibited MHCC97H cell lung metastasis (Fig. 5B). Importantly, CPT1C knockdown abolished miR-377-3p inhibitor-mediated promotion of MHCC97H cell lung metastasis. Taken together, these results demonstrate that the miR-377-3p/CPT1C axis regulates HCC metastasis in vivo.

### MiR-377-3p correlates with CPT1C expression and predicts clinical outcomes in HCC patients

**Fig. 6:**
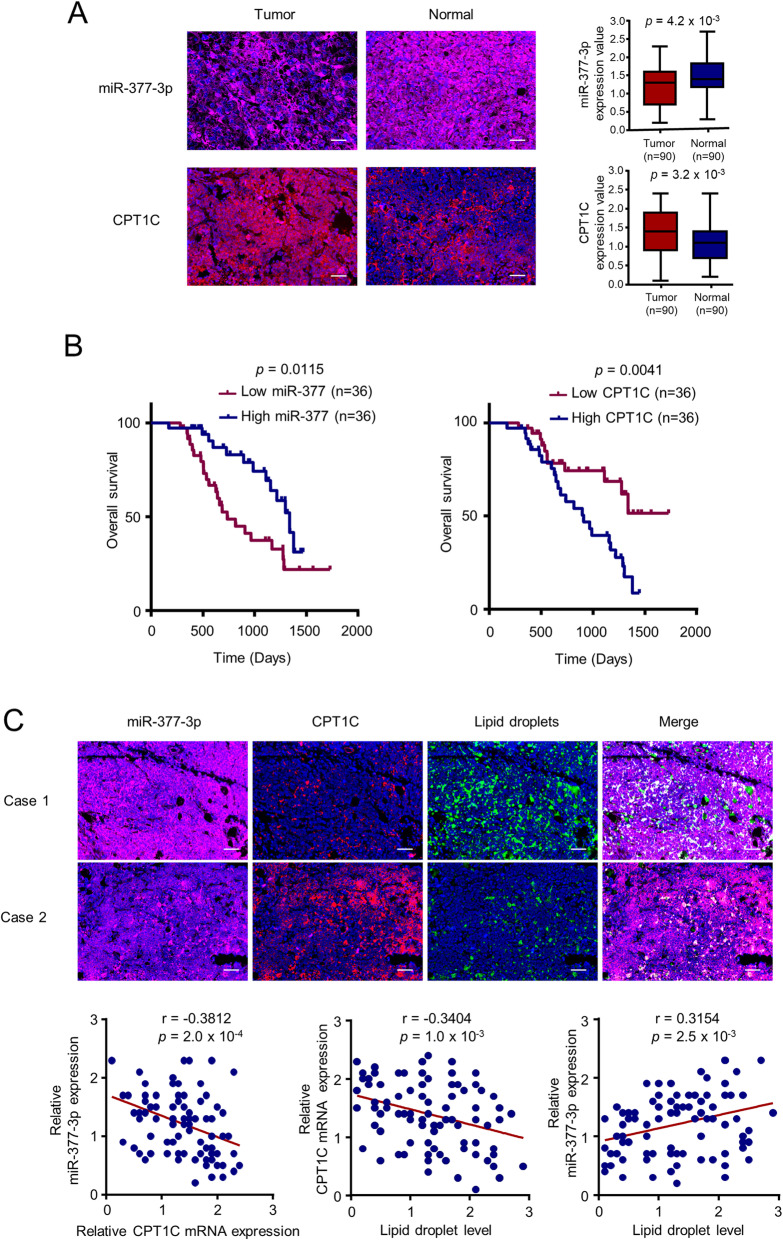
The clinical significance of miR-377-3p and CPT1C in HCC patients. **A** Representative miRNA in situ hybridization of miR-377-3p or immunohistochemical staining of CPT1C in 90 paired HCC tumor and adjacent normal tissues. Scale bar 50 μm. H scores of miR-377-3p or CPT1C expression between tumor and normal tissues were compared by Mann-Whitney *U* test. **B** Kaplan-Meier survival curves for overall survival of 72 HCC patients according to the relative expression of miR-377-3p and CPT1C. **C** Representative MISH of miR-377-3p or IHC staining of CPT1C or BODIPY 493/503 staining of lipid droplets in 90 HCC tissues. Correlation was determined between miR-377-3p and CPT1C expression (left panel), between CPT1C expression and lipid droplets (middle panel) and between miR-377-3p expression and lipid droplets (right panel) in 90 HCC patients. Scale bar 50 μm. The *p* value was generated using Spearman’s rank correlation test

To determine whether miR-377-3p and CPT1C expression had clinical significance, we collected 90 pairs of HCC tumor and adjacent normal samples. The results showed that miR-377-3p expression was significantly downregulated, and conversely, CPT1C expression was significantly upregulated in HCC patients, compared with normal tissues (Fig. 6A). The Kaplan-Meier survival analysis indicated that high miR-377-3p expression showed longer overall survival (OS) and high CPT1C expression exhibited shorter OS (Fig. 6B). Furthermore, we determined the miR-377-3p expression, CPT1C expression and lipid droplet level by MISH and IHC in 90 HCC samples we collected. Consistent with the results in vitro and in mice, there was an inverse correlation between miR-377-3p and CPT1C expression, as well as between CPT1C expression and lipid droplet level. In addition, there was a positive correlation between miR-377-3p and lipid droplet level in HCC patients (Fig. 6C). In conclusion, these data confirm that the miR-377-3p/CPT1C axis has a great clinical significance in HCC.

## Discussion

Lipids are a highly complex group of biomolecules that make up the building blocks of the structure and function of living cells and also function as signaling molecules and an energy source. Increasing evidence implicates that abnormal lipid metabolism, an important component of metabolism reprogramming, is found in various cancers and closely associated with tumor occurrence, development, invasion and metastasis. Fatty acid oxidation, mainly β-oxidation, is a catabolic process by which fatty acid molecules are broken down into acetyl-CoA units within the mitochondrial matrix, and energy is released from fatty acids meanwhile. Fatty acid oxidation is regulated in many ways to achieve a balance between energy production and expenditure. The rate-limiting step is the transport of fatty acyl-CoA to the mitochondria through the carnitine system, which is the most prominent aspect in fatty acid oxidation.

As the last member of CPT1 family, the location of CPT1C in cells remains controversial. Previous studies indicated that the ectopic expression of CPT1C in neuron cells and COS7 cells predominantly locates in endoplasmic reticulum [17, 33]. However, recent research showed that CPT1C located in both the nucleus and the cytoplasm in glioma cells [34]. CPT1C was also detected in mitochondria in PANC-1 cells [18]. Our data demonstrated that CPT1C colocalized with mitochondria in HCC cells. Therefore, the variation of CPT1C location may be due to the ectopic overexpression or cell line difference. Moreover, the carnitine acyltransferase activity and FAO function of CPT1C is still controversial. Many studies have indicated that CPT1C has minimal catalytic activity and binds malonyl-CoA to acts as an energy sensor, and enhances fatty acid oxidation [22, 35, 36]. In contrast, Sierra indicated that CPT1C had carnitine palmitoyltransferase activity, but did not participate in fatty acid oxidation [17]. Here, we show that CPT1C enhances the transport of palmitate into mitochondria, and increased FAO of HCC cells. These results suggest that CPT1C locates in mitochondria and transports FA into mitochondria for FAO, which is similar to CPT1A and CPT1B.

In our study, we demonstrated a critical role of the miR-377-3p/CPT1C axis in HCC for the first time. First, CPT1C expression was increased in HCC tissues compared to the paired adjacent normal tissues, and predicted a poor prognosis. Second, miR-377-3p was identified as a novel suppressor of CPT1C. Third, miR-377-3p decreased the rate of FAO and reduced cellular ATP production. Fourth, miR-377-3p inhibited tumor growth and metastasis by suppressing CPT1C-mediated fatty acid oxidation. These findings indicated that the miR-377-3p/CPT1C axis was a promising therapeutic target for cancer treatment.

MicroRNA (miRNA) is one of many types of non-coding RNA that can bind to the 3′-UTR of target mRNAs through an imperfect sequence to reduce their expression. MiR-377-3p has been reported to target multiple downstream genes to participate in tumor progression. For example, Zhan et al. showed that miR-377-3p repressed the proliferation, cycle progression, and migration of HCC cells through binding to the 3′-UTR of fibroblast growth factor receptor 1 (FGFR1) [37]. In colorectal cancer, miR-377-3p suppressed Wnt/ß-catenin signaling by directly targeting ZEB2 and XIAP, thus inhibiting the tumor progression [31]. In addition, miR-377-3p targeted LASP1 to decrease glioma cell proliferation and migration [38]. Our study declared that miR-377-3p reversely regulated CPT1C expression to function as a tumor suppressor, using bioinformatics predication and further experimental data verification. Moreover, miR-377-3p decreased fatty acid oxidation by inhibiting CPT1C and thereby modulated cell proliferation, migration and invasion in vitro and in vivo, suggesting another signaling pathway for miR-377-3p in regulating tumor occurrence and progression in HCC.

In the context of lipid metabolic modulation, inhibition of FAO is the most intensively investigated intervention. Etomoxir, an irreversible inhibitor of CPT1, prevents the formation of acyl carnitines that is necessary for the transport of long fatty acid chains into the mitochondria, leading to the reduction of FAO [39, 40]. Glioma cells treated with Etomoxir showed significantly slower growth rate, and less mitochondrial ATP production in vitro and in vivo [41]. Treatment of cells with Etomoxir by inhibiting FAO promoted lipid droplet abundance and decreased migration in triple-negative breast cancer [42]. In this study, HepG2 and MHCC97H cells treated with Etomoxir resulted in reduction of CPT1C-induced FAO and promotion of lipid droplets. Moreover, treatment with Etomoxir abolished the ability of miR-377-3p inhibitor to increase the proliferation, migration and invasion of these cells. In contrast, subsequent studies have indicated that circulating free acetate may be an important carbon source to generate acetyl-CoA, which promotes lipid synthesis and tumor growth [43]. In ACBP KD glioma cell, acetate supplementation rescued the proliferation rates to control cells [41]. In HepG2 liver cancer cell, acetate activated ACACA and FASN expression and increased de novo lipid synthesis under hypoxia, thus promoting cell survival [44]. Here, our results demonstrated that acetate addition attenuated the suppression of miR-377-3p-mediated cell proliferation, migration, and invasion. Taken together, we declared that the miR-377-3p/CPT1C axis modulates HCC growth and metastasis through fatty acid oxidation.

## Conclusion

In conclusion, this is the first study to elucidate the location and biological function of CPT1C in HCC. CPT1C locates in mitochondria. MiR-377-3p targets CPT1C to reduce its expression, and inhibits cell proliferation, migration, invasion, and metastasis through regulating fatty acid oxidation in vitro and in vivo. In HCC tissues, miR-377-3p exhibits low expression and CPT1C shows high expression, and the two are negatively correlated. Moreover, miR-377-3p and CPT1C predict a good and worse prognosis, respectively. These lines of evidence put forward potential strategies by which targeting the miR-377-3p/CPT1C axis may provide a therapeutic window for cancer treatment.

## Supplementary Information

**Additional file 1:.** Figure S1. MiR-377-3p inhibits CPT1C expression.

**Additional file 2:.** Figure S2. MiR-377-3p regulates FAO of lipid metabolism through CPT1C.

**Additional file 3.** Figure S3. MiR-377-3p suppresses proliferation, migration and invasion through inhibition of CPT1C expression in HCC cells.

**Additional file 4:.** Figure S4. The miR-377-3p/CPT1C axis regulates the proliferation, migration and invasion of HCC cells mainly through FAO.

**Additional file 5:.** Figure S5. The miR-377-3p/CPT1C axis regulates the proliferation, migration and invasion of HCC cells mainly through FAO.

**Additional file 6:.** Table S1 Primes sequence for shRNA, miRNA mimics, probe and qRT-PCR

## Data Availability

All data needed to evaluate the conclusions in the paper are present in the paper and/or the Supplementary Materials. Additional data related to this paper may be requested from the authors. Declarations Ethics approval and consent to participate This study was approved by the Institutional Ethical Review Committee of Chinese PLA General Hospital (Beijing, China). All animal studies were approved by the Institutional Animal Care Committee of Beijing Institute of Biotechnology (Beijing, China).

## Abbreviations

CPT1C: Carnitine palmitoyltransferase 1C
miRNA: microRNA
HCC: Hepatocellular carcinoma
FAO: Fatty acid oxidation
UTR: Untranslated region
ATCC: American Type Culture Collection
DMEM: Dulbecco’s modified Eagle’s medium
FBS: Fetal bovine serum
IHC: Immunohistochemistry
WT: Wild type
MUT: Mutant
HRP: Horseradish peroxidase
qRT-PCR: Quantitative real-time PCR
