# Supplementary figures and images for: MicroRNA-377-3p inhibits hepatocellular carcinoma growth and metastasis through negative regulation of CPT1C-mediated fatty acid oxidation
Source: Cancer Metab. 2022 Jan 20;10:2. doi: 10.1186/s40170-021-00276-3 (PMC8772112; doi:10.1186/s40170-021-00276-3)

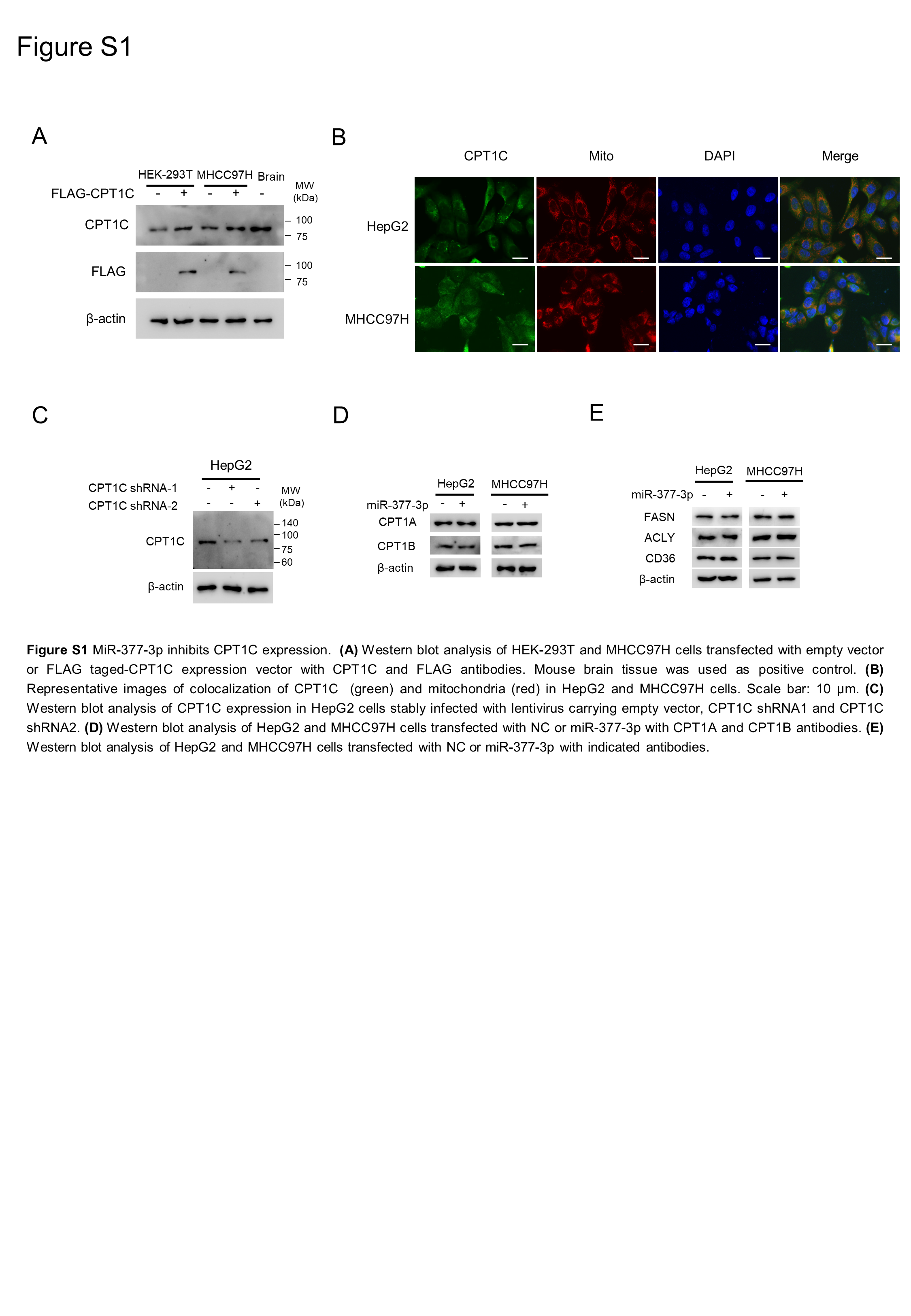

Supplement: Supplementary file 1 — Additional file 1:. Figure S1. MiR-377-3p inhibits CPT1C expression. [file 40170_2021_276_MOESM1_ESM.tif]

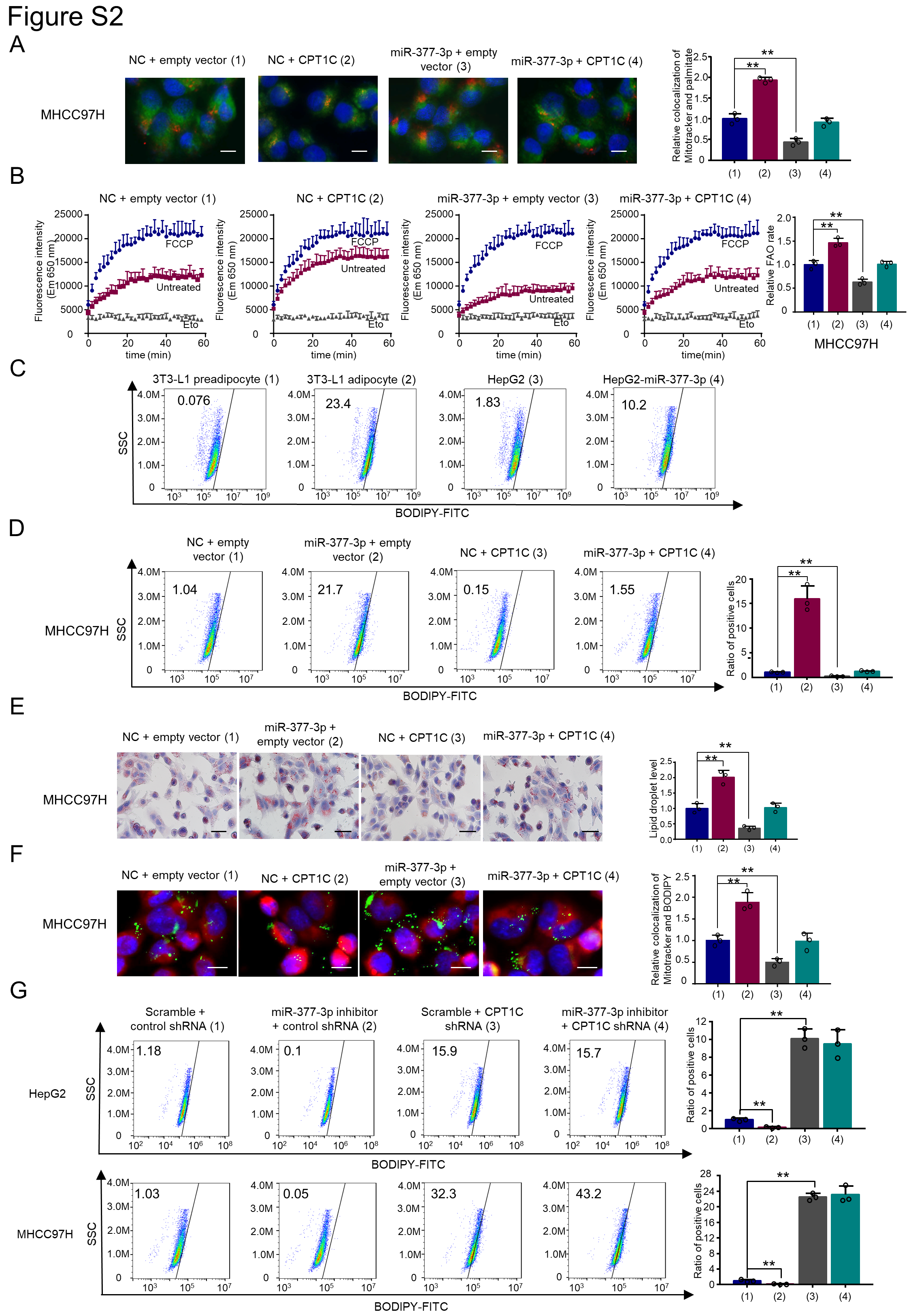

Supplement: Supplementary file 2 — Additional file 2:. Figure S2. MiR-377-3p regulates FAO of lipid metabolism through CPT1C. [file 40170_2021_276_MOESM2_ESM.zip › 40170_2021_276_MOESM2_ESM/Figure S2/Figure S2-1.tif]

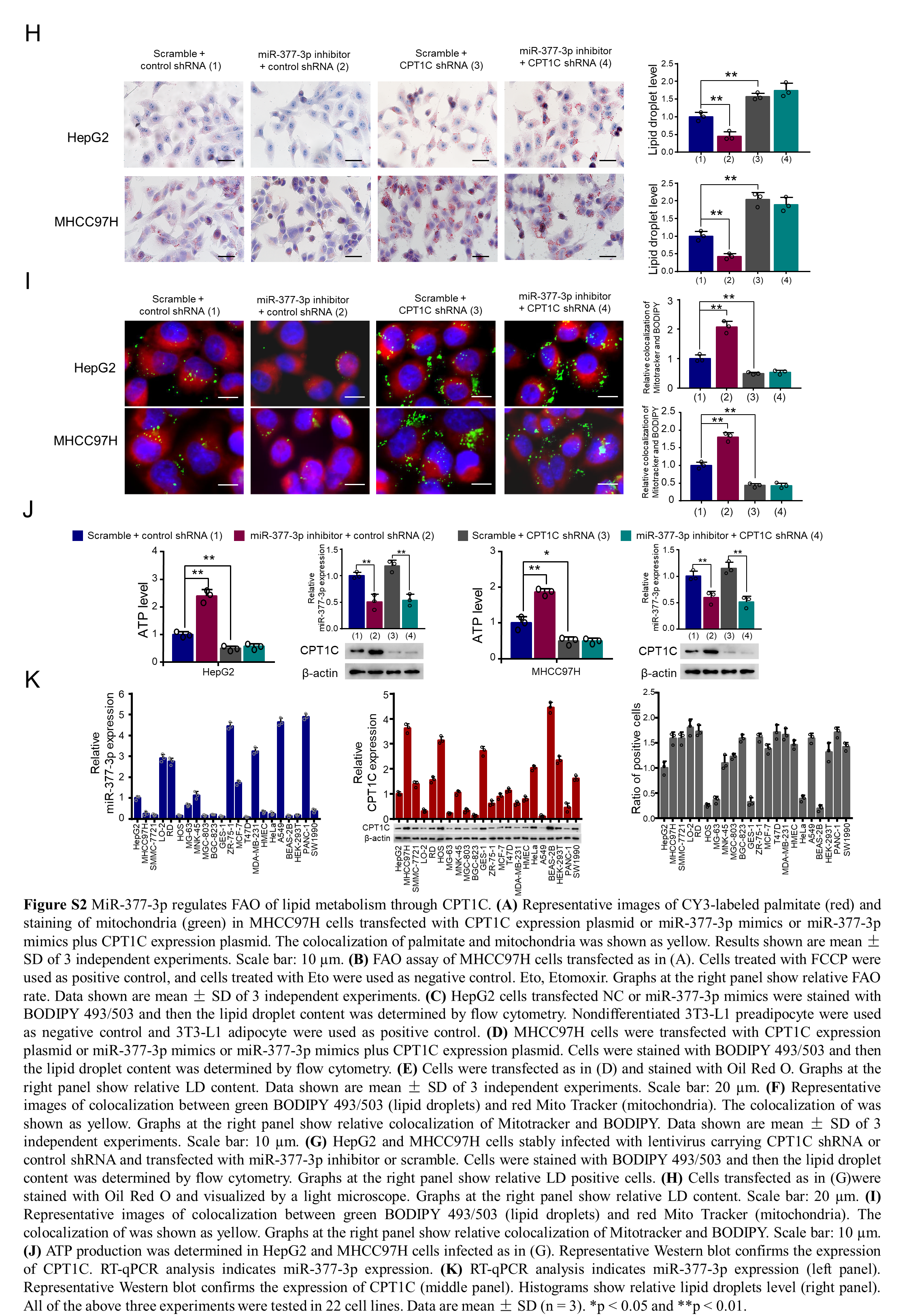

Supplement: Supplementary file 2 — Additional file 2:. Figure S2. MiR-377-3p regulates FAO of lipid metabolism through CPT1C. [file 40170_2021_276_MOESM2_ESM.zip › 40170_2021_276_MOESM2_ESM/Figure S2/Figure S2-2.tif]

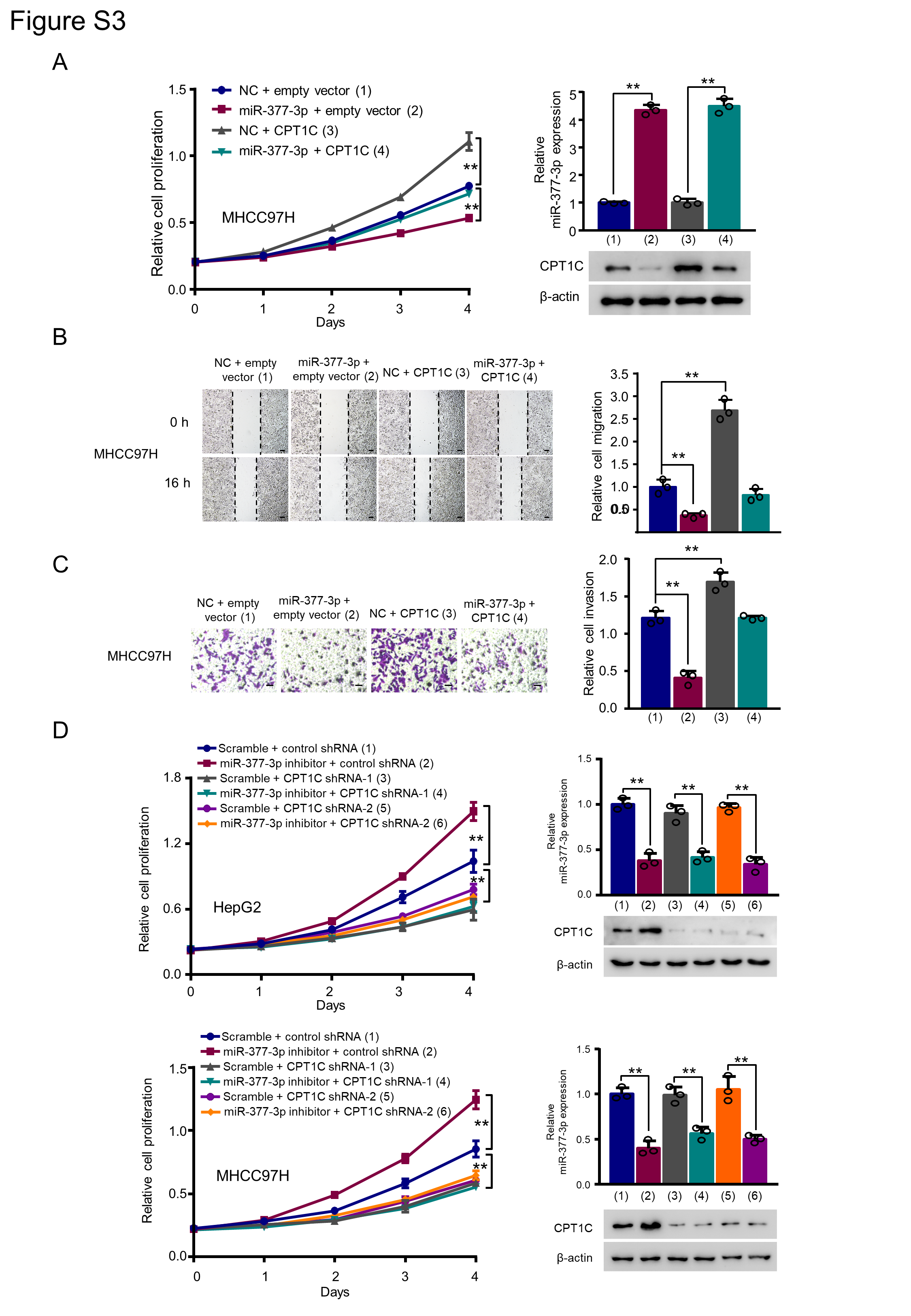

Supplement: Supplementary file 3 — Additional file 3. Figure S3. MiR-377-3p suppresses proliferation, migration and invasion through inhibition of CPT1C expression in HCC cells. [file 40170_2021_276_MOESM3_ESM.zip › 40170_2021_276_MOESM3_ESM/Figure S3/Figure S3-1.tif]

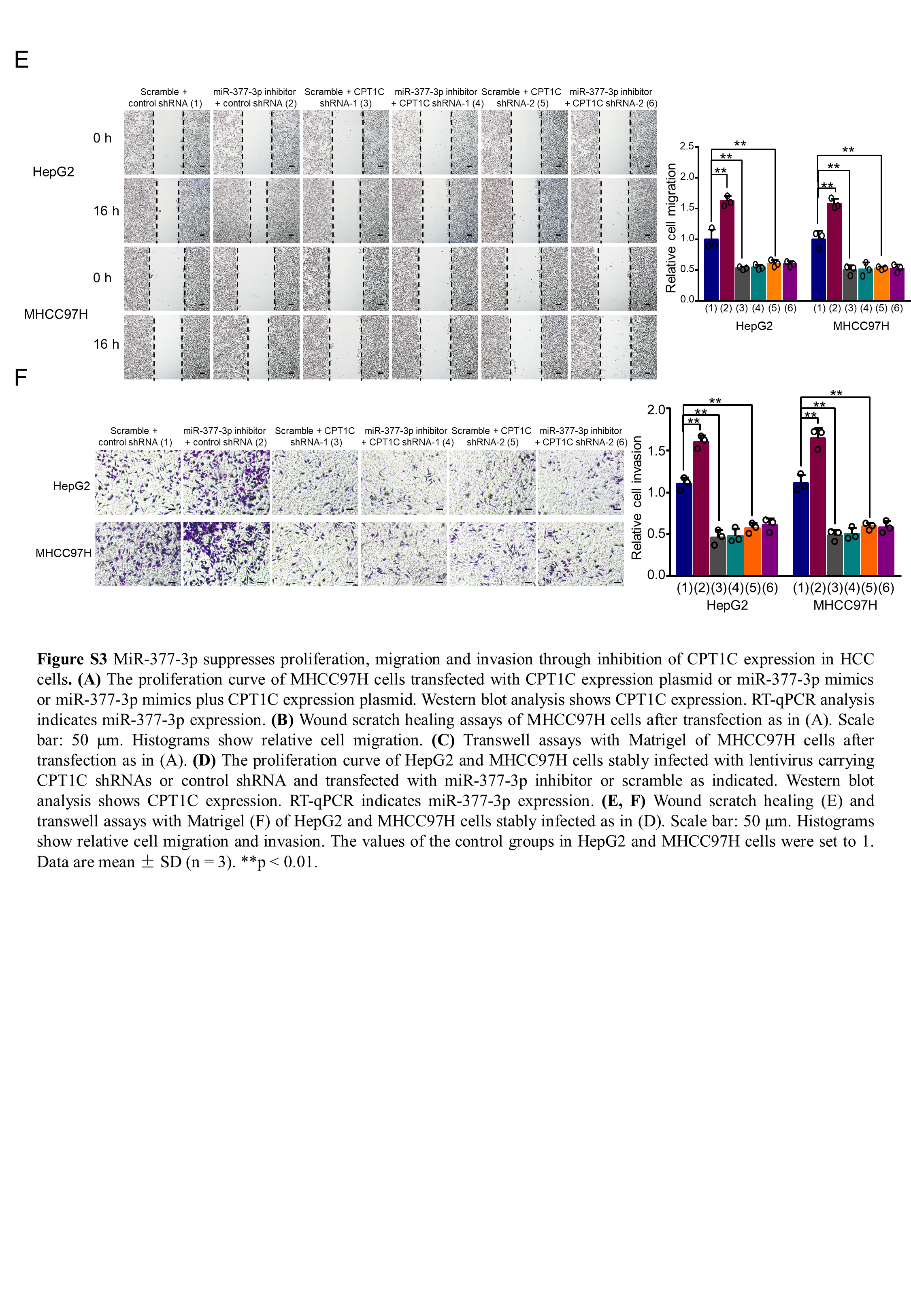

Supplement: Supplementary file 3 — Additional file 3. Figure S3. MiR-377-3p suppresses proliferation, migration and invasion through inhibition of CPT1C expression in HCC cells. [file 40170_2021_276_MOESM3_ESM.zip › 40170_2021_276_MOESM3_ESM/Figure S3/Figure S3-2.tif]

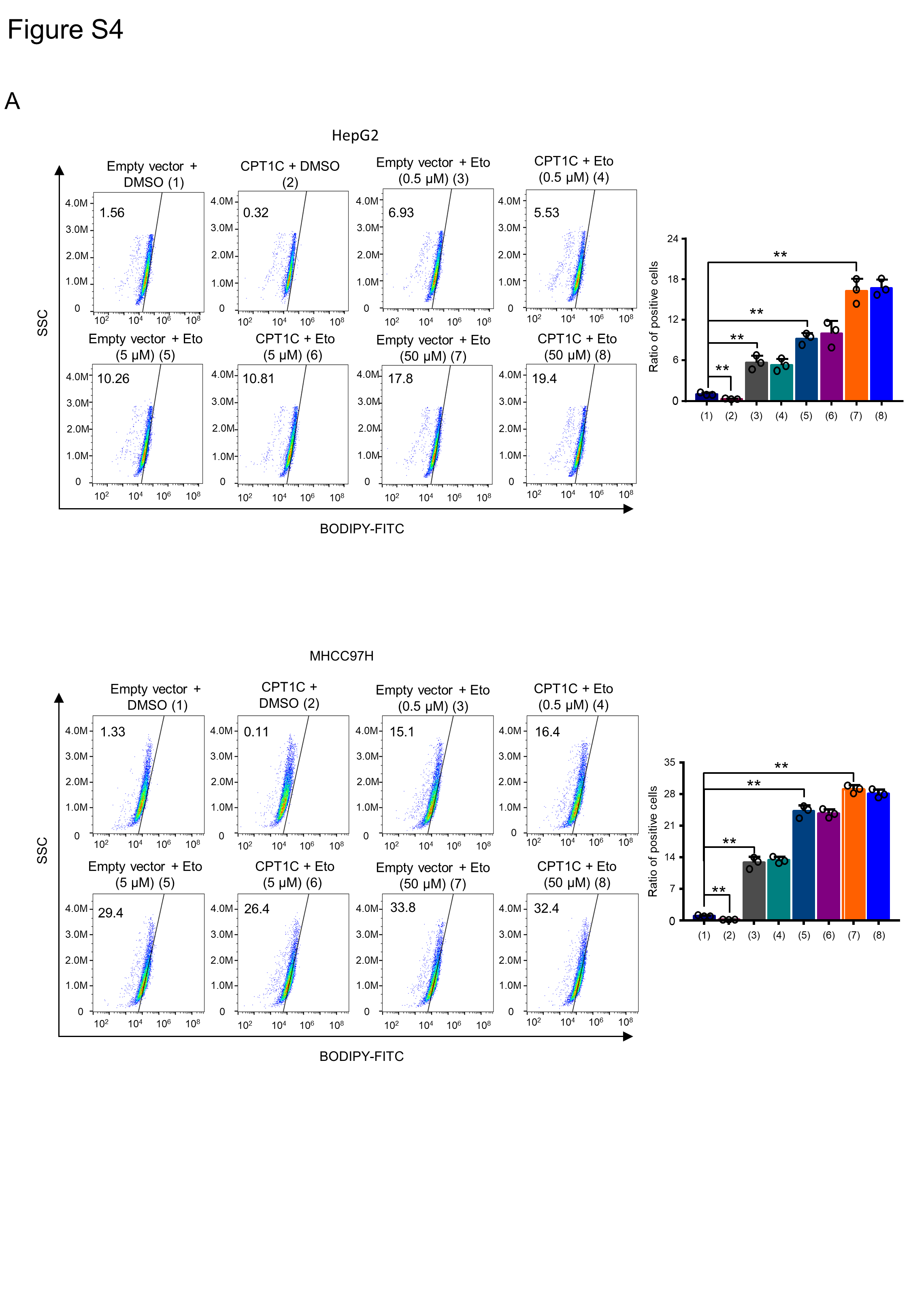

Supplement: Supplementary file 4 — Additional file 4:. Figure S4. The miR-377-3p/CPT1C axis regulates the proliferation, migration and invasion of HCC cells mainly through FAO. [file 40170_2021_276_MOESM4_ESM.zip › 40170_2021_276_MOESM4_ESM/Figure S4/Figure S4-1.tif]

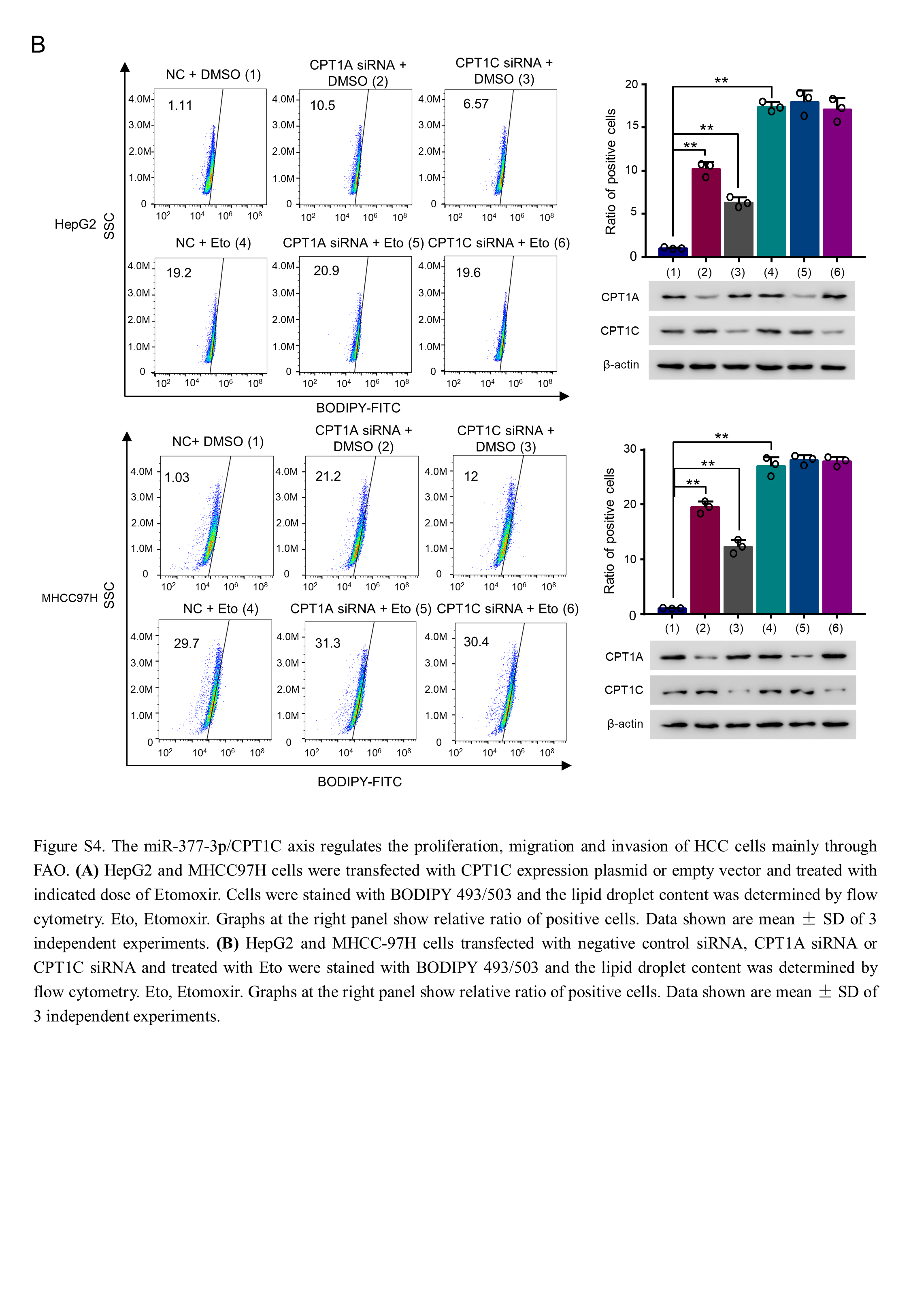

Supplement: Supplementary file 4 — Additional file 4:. Figure S4. The miR-377-3p/CPT1C axis regulates the proliferation, migration and invasion of HCC cells mainly through FAO. [file 40170_2021_276_MOESM4_ESM.zip › 40170_2021_276_MOESM4_ESM/Figure S4/Figure S4-2.tif]

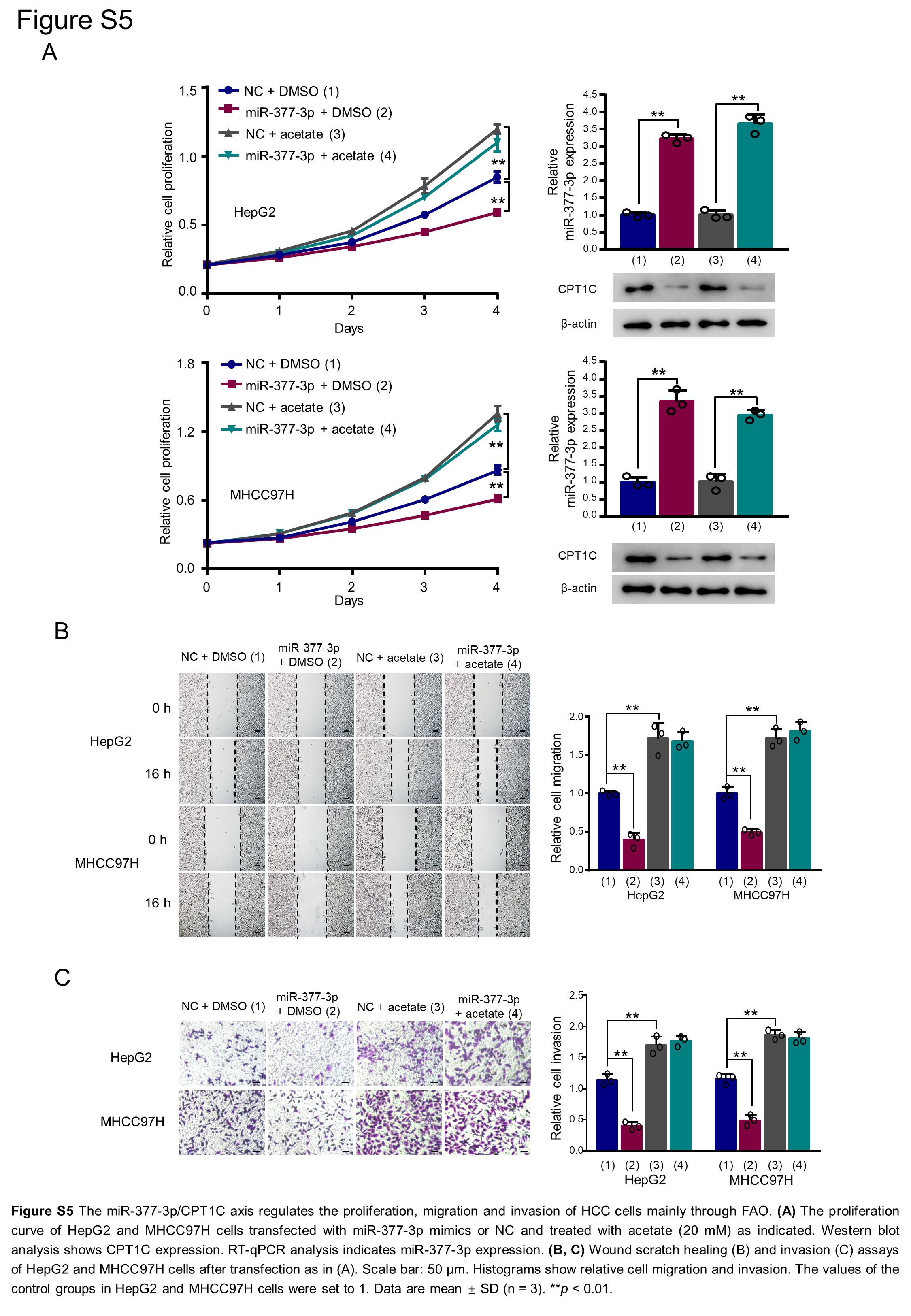

Supplement: Supplementary file 5 — Additional file 5:. Figure S5. The miR-377-3p/CPT1C axis regulates the proliferation, migration and invasion of HCC cells mainly through FAO. [file 40170_2021_276_MOESM5_ESM.tif]
