# Supplementary material for: MicroRNA-377-3p inhibits hepatocellular carcinoma growth and metastasis through negative regulation of CPT1C-mediated fatty acid oxidation
Source: Cancer Metab. 2022 Jan 20;10:2. doi: 10.1186/s40170-021-00276-3 (PMC8772112; doi:10.1186/s40170-021-00276-3)
Supplement: Supplementary file 6 — Additional file 6:. Table S1 Primes sequence for shRNA, miRNA mimics, probe and qRT-PCR [file 40170_2021_276_MOESM6_ESM.docx]

| Primer name | Sequences (5’→3’) |
| --- | --- |
| Control shRNA: | 5’-TTCTCCGUUC GTGTCUCGT-3’ |
| CPT1C shRNA-1： | 5’-GAAATCCGCTGATGGTGAA-3’ |
| CPT1C shRNA-2: | 5’-GACAAATCCTTCACCCTAA-3’ |
| CPT1A siRNA: | 5’-GGUGGUUUGACAAGUCGUUCA-3’ |
| CPT1C siRNA: | 5’-GAAAUCCGCUGAUGGUGAA-3’ |
| miR-339-5p mimics： | 5’-UCCCUGUCCUCCAGGAGCUCACG-3’ |
| miR-432-5p mimics： | 5’-UCUUGGAGUAGGUCAUUGGGUGG-3’ |
| miR-592 mimics： | 5’-UUGUGUCAAUAUGCGAUGAUGU-3’ |
| miR-342-3p mimics： | 5’-UCUCACACAGAAAUCGCACCCGU-3’ |
| miR-4267 mimics： | 5’-UCCAGCUCGGUGGCAC-3’ |
| miR-377-3p mimics： | 5’-AUCACACAAAGGCAACUUUUGU-3’ |
| miR-377-3p inhibitor： | 5’-ACAAAAGUUGCCUUUGUGUGAU-3’ |
| miR-377-3p probe: | 5’-TAGTGTGTTTCCGTTGAAAACA-3’ |
| U6 probe: | 5’-GAACGCTTCACGAATTTGCGTG  TCATCCTTGCGCA-3’ |
| Scramble probe | 5’-GTGTAACACGTCTATACGCCCA-3’ |
| CPT1C-3’UTR(WT) F： | 5’-CTCCTTCCAGCAGGCAGC-3’ |
| CPT1C-3’UTR(WT) R： | 5’-CCCAGCTCACACATCTTTAT-3’ |
| CPT1C-3’UTR(Mut) F： | 5’-CTCCTTCCAGCAGGCAGC-3’ |
| CPT1C-3’UTR(Mut) R： | 5’-CCCAGCCTGT GTGTCTTTAT-3’ |
| miR-377-3p for RT-qPCR: | 5’-CTCAACTGGTGTCGTGGAGTCGG  CAATTCAGTTGAGCACAAAA-3’ |
| U6 for RT-qPCR: | 5’-AACGCTTCACGAATTTGCGT-3’ |
| miR-377-3p F: | 5’-CAGG ATCACACAAAGGCAAC-3’ |
| miR-377-3p R: | 5’-CTCAACTGGTGTCGTGGAG-3’ |
| U6 F： | 5’-CTCGCTTCGGCAGCACA-3’ |
| U6 R： | 5’-AACGCTTCACGAATTTGCGT-3’ |
| CPT1C F： | 5’-GGATGGCACTGAAGAGGAAA-3’ |
| CPT1C R： | 5’-TCCTGGAAAAGGCATCTCTC-3’ |
| β-actin F: | 5’-TCGTGCGTGACATTAAGGAG-3’ |
| β-actin R: | 5’-ATGCCAGGGTACATGGTGGT-3’ |

**Table S1 Primes sequence for shRNA, miRNA mimics, probe and qRT-PCR**
